# The diagnostic value of metagenomics next-generation sequencing in HIV-infected patients with suspected pulmonary infections

**DOI:** 10.3389/fcimb.2024.1395239

**Published:** 2024-05-07

**Authors:** Mingjie Hou, Yanli Wang, Haizhen Yuan, Yuwei Zhang, Xia Luo, Ningbo Xin, Qingxia Zhao

**Affiliations:** Department of Infectious Diseases, The Sixth People Hospital of Zhengzhou, Zhengzhou, China

**Keywords:** people living with HIV (PLHIV), pulmonary infection, metagenomic next-generation sequencing (mNGS), diagnosis, medicine

## Abstract

**Background:**

Traditional microbiological detection methods used to detect pulmonary infections in people living with HIV (PLHIV) are usually time-consuming and have low sensitivity, leading to delayed treatment. We aimed to evaluate the diagnostic value of metagenomics next-generation sequencing (mNGS) for microbial diagnosis of suspected pulmonary infections in PLHIV.

**Methods:**

We retrospectively analyzed PLHIV who were hospitalized due to suspected pulmonary infections at the sixth people hospital of Zhengzhou from November 1, 2021 to June 30, 2022. Bronchoalveolar lavage fluid (BALF) samples of PLHIV were collected and subjected to routine microbiological examination and mNGS detection. The diagnostic performance of the two methods was compared to evaluate the diagnostic value of mNGS for unknown pathogens.

**Results:**

This study included a total of 36 PLHIV with suspected pulmonary infections, of which 31 were male. The reporting period of mNGS is significantly shorter than that of CMTs. The mNGS positive rate of BALF samples in PLHIV was 83.33%, which was significantly higher than that of smear and culture (44.4%, P<0.001). In addition, 11 patients showed consistent results between the two methods. Futhermore, mNGS showed excellent performance in identifying multi-infections in PLHIV, and 27 pathogens were detected in the BALF of 30 PLHIV by mNGS, among which 15 PLHIV were found to have multiple microbial infections (at least 3 pathogens). Pneumocystis jirovecii, human herpesvirus type 5, and human herpesvirus type 4 were the most common pathogen types.

**Conclusions:**

For PLHIV with suspected pulmonary infections, mNGS is capable of rapidly and accurately identifying the pathogen causing the pulmonary infection, which contributes to implement timely and accurate anti-infective treatment.

## 1 Introduction

HIV infection leads to systemic destruction of T-cells and reduction in cell-mediated immunity in the human body, thereby increasing the risk of opportunistic infections (Yuan et al., 2022). Of all the organs in the human body, the lungs are the most prone to infections by microorganisms such as viruses, bacteria, fungi, and parasites (Gu et al., 2019; Cribbs et al., 2020). Consequently, the prompt and accurate diagnosis of pulmonary infections is vital for devising preventative and therapeutic strategies to enhance pulmonary health and decrease mortality rates in people living with HIV (PLHIV).

Next-generation sequencing (NGS), also known as high-throughput or massively parallel sequencing, is a technological category that enables the concurrent independent sequencing of a range from thousands to billions of DNA segments (Gu et al., 2019). Metagenomics Next-Generation Sequencing (mNGS) represents the utilization of NGS technology for the purpose of clinical microbial identification. Conventional methods for microbial detection are limited to the identification of approximately 40% of pathogens and often require a significant amount of time. However, mNGS offers numerous benefits including increased speed, unbiased sampling, and a wide range of pathogen detection (Gu et al., 2019; Guo et al., 2021).

Prompt recognition of pathogens plays a critical role in clinical treatment, notably in the precise utilization of antibiotics. In situations where a definitive microbiological diagnosis is lacking, patients suffering from HIV along with lung infections are commonly subjected to empirical therapy involving broad-spectrum antibiotics during the initial treatment phase to relieve symptoms. This could potentially lead to the overutilization of broad-spectrum antibiotics (Duan et al., 2021; Mao et al., 2022). Thus, mNGS could significantly aid in the prompt microbiological diagnosis and accurate treatment of individuals who may have infections. While there are some studies on the use of mNGS for detecting lung infections in the general patients, there is scant information on its application in PLHIV, who usually have compromised immunity and advanced disease stages. Moreover, existing studies have focused on diagnosing single infections rather than multiple infections, which are quite common in PLHIV (Mao et al., 2022; Niu et al., 2023).

In this study, we aimed to evaluate the performance of mNGS in diagnosing lung infections in PLHIV by comparing the results of mNGS, including precise classification, positive detection rate, and multi-infections, with those of Conventional Microbiological Tests (CMTs). The results provide evidence for the clinical diagnosis and treatment of lung infections in PLHIV.

## 2 Materials and methods

### 2.1 Materials and testing

The study retrospectively analyzed 36 PLHIV hospitalized to the infectious diseases department of the sixth people hospital of Zhengzhou. Inclusion criteria including 1) HIV infection diagnosis, 2) hospitalized in infectious diseases department of the sixth people hospital of Zhengzhou between November 1, 2021 to June 30, 2022 for high suspicion of infection in the lungs, 3) received test for suspicious pulmonary infection using both traditional microbiology and mNGS. All PLHIV involved in the study were briefed about the research and gave their consent via a signed written informed consent document before the commencement of the study. The Ethics Committee of the sixth people hospital of Zhengzhou granted approval for this study, under the ethics code IEC-KY-2022-001.

#### 2.1.1 Collection of bronchoalveolar lavage fluid via painless fiberoptic bronchoscopy

BALF samples were gathered by bronchoscopists based on standard procedures using painless fiberoptic bronchoscopy (PENTAX EG-2490K with an outer diameter of 7.5mm, PENTAX EG27-i10 with an outer diameter of 9.0mm, PENTAX EG29-i10 with an outer diameter of 9.8mm). The collected BALF samples were used for subsequent mNGS and CMTs, which include cultivation, smear, and PCR detection.

#### 2.1.2 Metagenomic next-generation sequencing

##### 2.1.2.1 DNA extraction

The DNA extraction process followed the protocol of the Bacterial Genome DNA Extraction Kit (Hangzhou Jieyi Biotechnology Co., LTD., MD049). The concentration of the extracted DNA was determined by the Thermo Nanodrop 2000 Ultramicro Spectrophotometer (Themo Scientmc, USA, NanoDrop 2000) and stored at -20°C. The extracted DNA is segmented into approximately 300bp fragments using Biological sample homogenizer (Hangzhou Jieyi Biotechnology Co., LTD., BSP-060). Subsequently, the TruSeq Nano DNA LT library Prep Kit (Hangzhou Jieyi Biotechnology Co., LTD., MD001) was used to prepare the sequencing library, which was then checked with an Agilent Bioanalyzer.

##### 2.1.2.2 High-throughput sequencing with Illumina Hiseq500

Quantification of the approved library was performed using the Promega Quantifluor Fluorescent Quantification System (Xi **‘**an Tianlong Technology Co., LTD., Gentier 96R) and the Quant-iT PicoGreen dsDNA Assay Kit (Hangzhou Jieyi Biotechnology Co., LTD., MD004). After gradient dilution, the approved sequencing library was mixed according to the required sequencing volume at the corresponding ratio, and denatured into single strands using NaOH for online sequencing. PE50 sequencing was performed using the Illumina Hiseq500 sequencer (Illumina, Nextseq 550DX).

#### 2.1.3 Conventional microbial tests

##### 2.1.3.1 Cultivation and smear

The BALF samples were subjected to routine culture and smear. After standard specimen processing, they were inoculated onto culture plates. Following incubation in a Panasonic MCO-18AC carbon dioxide incubator, colony counts were performed. The number of bacteria = the number of colonies with the same morphology × 100 CFU/mL. If no pathogenic microorganisms grow within 48 hours, it was identified as negative. If the colony count of pathogenic bacteria or conditional pathogenic bacteria reaches 104 CFU/mL or more, it was identified as positive. Once cultivation was finished, cells from the specimen were centrifuged and smeared. After air-drying and fixing with methanol, Gram staining was carried out with a detection sensitivity of 105 per mL. The identification of cultured microbial strains followed the instructions provided by the mass spectrometer (Manufacturer: Bruker Daltonics; Model: Microflex LT/SH).

##### 2.1.3.2 Polymerase chain reaction

A 0.5 mL BALF sample was combined with 0.5 µL of sodium hydroxide solution and left to stand at room temperature for 40 minutes. The mixture was then centrifuged at 10,000 rpm for 5 minutes. The supernatant was discarded and the residue was washed with saline. An extraction solution (50 µL) was added and the mixture was shaken. It was then boiled at 100°C for 10 minutes and centrifuged at 4°C for another 5 minutes before the template was amplified. The TB-DNA template was prepared by adding 0.2 µL of Taq enzyme and 15 µL of reaction mixture, sealing with 20 µL of paraffin oil, and adding 5 µL of supernatant. The mixture was then amplified at speeds of 10,000 r/min at temperatures of 55, 72, and 90 °C for durations of 30, 30, and 60 seconds respectively over the course of 35 cycles. Electrophoresis was performed on 20 mL of PCR amplification solution, and a positive result was indicated by the appearance of an orange-yellow fluorescent band.

### 2.2 Statistical analysis

Continuous variables are represented by mean ± standard deviation (SD) or median (25th, 75th percentile), while categorical variables are expressed as numbers (percentages). The Chi-square test, McNemar’s test, one-way ANOVA test or Fisher’s exact test are used to compare the diagnostic performance of mNGS and CMTs. P-value of less than 0.05 was considered significant. Analysis was performed by SPSS 26.0.

## 3 Results

### 3.1 Patient characteristics

A total of 36 PLHIV were included in this study (**Table 1**). The majority of the participants were male (31/36), with an average age of 44.25 ± 16.27 years. The results of the laboratory tests were as follows: CD4+T cell count was 50.50 (16.50, 187.75) cells/μL; CD4/CD8 ratio was 0.10 (0.03, 0.40); and white blood cell count was 5.02 (3.48, 8.13) x109/L. And almost half (41.67%) of our participants can not be detected HIV viral load.

**Table 1:** Clinical characteristics of 36 participants.

Characteristics	Value
Age (years)	44.25 ± 16.27
Gender (female vs. male)	31/5
CD4^+^T cell count, cells/μL	50.50 (16.50, 187.75)
CD4/CD8	0.10 (0.03, 0.40)
White blood cell count, 10^9^/L	5.02 (3.48, 8.13)
HIV viral load (Copies/ml)	
Can not be detected	15 (41.67)
<1000	7 (19.44)
≥1000	14 (38.89)

### 3.2 Diagnostic performance of mNGS and CMTs

#### 3.2.1 Reporting period

**Figure 1:**
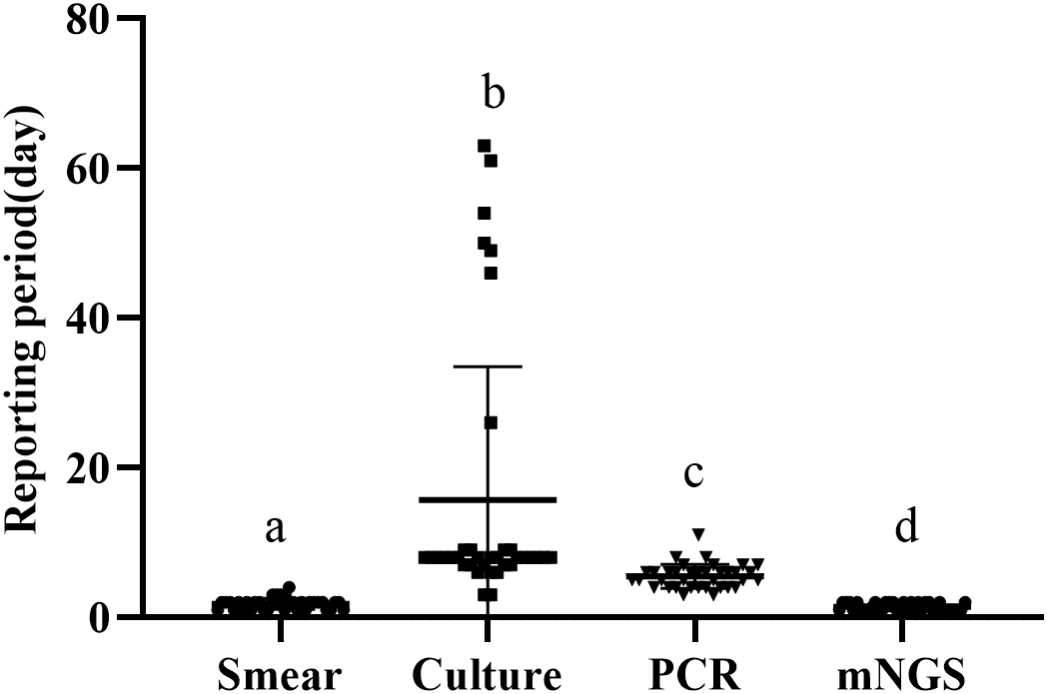
Reporting period of smear, culture, PCR and metagenomic next generation sequencing (mNGS) (a-d) if the superscript letters are different, it means there are significant differences between them.

We define the reporting period as the reporting time minus the inspection time, and compare the length of the reporting period of the four methods. The average reporting period of smear was 1.83 ± 0.73 days, that of culture was 15.67 ± 17.57 days, that of PCR was 5.50 ± 1.57 days, and that of mngs was 1.42 ± 0.49 days (**Figure 1**). There were significant differences in the reporting period of the four methods (p<0.001). Further comparison of the four methods showed that the reporting period of mNGS was the shortest, followed by smears, then PCR, and finally cultures with the longest reporting period.

#### 3.2.2 Pathogen detection by mNGS relative to CMTs

We compared the differences in pathogenic microorganisms among 36 PLHIV using two different detection methods (**Table 2**). A total of 30 pathogens were identified among the 36 infected individuals using mNGS, including 8 fungi (29/74), 10 viruses (31/74), 10 bacteria (11/74), and 2 mycoplasmas (3/74). In contrast, CMTs detected only 13 pathogens, comprising just 6 fungi (6/22), 6 bacteria (10/22), and 1 virus (6/22).

**Table 2:** Distribution of pathogens in 36 PLHIV based on Metagenomic next-generation sequencing and conventional microbiological tests.

ID	CMTs		mNGS	
	Microbial classification	positive results	Microbial classification	positive results
1	Pathogen negative		DNA virus	Primate erythroid parvovirus type 1
2	Pathogen negative		Fungi	Pneumocystis jiroveci
			Fungi	Aspergillus nidularis
			Fungi	Cryptococcus neoformans Grubii variant
3	Pathogen negative		DNA virus	Epstein-Barr virus
4	Pathogen negative		Pathogen negative	
5	Fungi	Candida albicans	Mycoplasma	Mycoplasma hominis
			DNA virus	Human cytomegalovirus
			RNA virus	Nasovirus type C
			Fungi	Pneumocystis jiroveci
			Fungi	Candida albicans
6	Pathogen negative		Gram-positive bacteria	Staphylococcus aureus
7	Pathogen negative		Fungi	Pneumocystis jiroveci
8	Pathogen negative		Fungi	Aspergillus flavus/Aspergillus oryzae
			DNA virus	Epstein-Barr virus
			DNA virus	Human cytomegalovirus
9	Pathogen negative		Pathogen negative	
10	DNA virus	Human cytomegalovirus	Fungi	Pneumocystis jiroveci
			Fungi	Aspergillus flavus/Aspergillus oryzae
			DNA virus	Herpes simplex virus-1
			DNA virus	Epstein-Barr virus
			DNA virus	Human cytomegalovirus
11	DNA virus	Human cytomegalovirus	Fungi	Pneumocystis jiroveci
			DNA virus	Epstein-Barr virus
			DNA virus	Human cytomegalovirus
12	DNA virus	Human cytomegalovirus	DNA virus	Epstein-Barr virus
			DNA virus	Human cytomegalovirus
13	Pathogen negative		DNA virus	Epstein-Barr virus
14	Gram-positive bacteria	G+cocci	Fungi	Aspergillus fumigata
	Gram-positive bacteria	G+ bacilli		
15	Gram-positive bacteria	G+cocci	Fungi	Pneumocystis jiroveci
16	DNA virus	Human cytomegalovirus	Fungi	Pneumocystis jiroveci
			DNA virus	Human cytomegalovirus
			DNA virus	Molluscum contagiosum virus
17	Fungi	Marneffei basket fungus	Fungi	Pneumocystis jiroveci
			RNA virus	Human coronavirus OC43
			Fungi	Marneffei basket fungus
18	Pathogen negative		Fungi	Aspergillus fumigata
19	Pathogen negative		Pathogen negative	
20	Pathogen negative		Fungi	Pneumocystis jiroveci
			DNA virus	Human cytomegalovirus
			RNA virus	Nasovirus type B
21	Pathogen negative		DNA virus	Epstein-Barr virus
			DNA virus	Molluscum contagiosum virus
			Mycoplasma	Ureaplasma urealyticum
			Gram-positive bacteria	Mycobacterium avium complex
22	Pathogen negative		Fungi	Pneumocystis jiroveci
23	Gram-negative bacteria	Raoultella ornithinolytica	Fungi	Pneumocystis jiroveci
	DNA virus	Human cytomegalovirus	DNA virus	Human cytomegalovirus
			RNA virus	Nasovirus type C
24	Fungi	Pneumocystis jiroveci	Fungi	Pneumocystis jiroveci
	Fungi	Aspergillus fumigata	Mycoplasma	Mycoplasma hominis
	Gram-positive bacteria	Mycobacterium Tuberculosis	RNA virus	Nasovirus type C
			RNA virus	Human parainfluenza virus type 3
25	Pathogen negative		Pathogen negative	
26	Fungi	Aspergillus flavus complex	Fungi	Pneumocystis jiroveci
	Fungi	aspergillus flavus	DNA virus	Human cytomegalovirus
	DNA virus	Human cytomegalovirus	Fungi	Aspergillus flavus/Aspergillus oryzae
			Fungi	Fusarium verticillioide
27	Gram-negative bacteria	Pseudomonas aeruginosa	Gram-negative bacteria	Pseudomonas aeruginosa
28	Pathogen negative		Pathogen negative	
29	Pathogen negative		Fungi	Pneumocystis jiroveci
			DNA virus	Human cytomegalovirus
30	Pathogen negative		Pathogen negative	
31	Gram-positive bacteria	Mycobacterium Tuberculosis	Gram-negative bacteria	Acinetobacter baumannii
			Gram-positive bacteria	Mycobacterium tuberculosis complex
32	Pathogen negative		Fungi	Pneumocystis jiroveci
			Fungi	Aspergillus flavus/Aspergillus oryzae
			RNA virus	Human coronavirus NL63
			Gram-positive bacteria	Staphylococcus haemolyticus
33	Gram-negative bacteria	Klebsiella pneumoniae	Fungi	Aspergillus flavus/Aspergillus oryzae
			Gram-negative bacteria	Moraxella catarrhalis
			Gram-negative bacteria	Klebsiella pneumoniae
34	Pathogen negative		Fungi	Aspergillus fumigata
			DNA virus	Epstein-Barr virus
			DNA virus	Human cytomegalovirus
35	Pathogen negative		Fungi	Pneumocystis jiroveci
			DNA virus	Human cytomegalovirus
36	Gram-negative bacteria	Pseudomonas aeruginosa	Gram-positive bacteria	Streptococcus pneumoniae
	Gram-positive bacteria	Mycobacterium Tuberculosis	Gram-positive bacteria	Mycobacterium avium complex
			Gram-positive bacteria	Streptococcus mitis

mNGS, Metagenomic next-generation sequencing; CMTs, Conventional microbiological tests.

**Figure 2:**
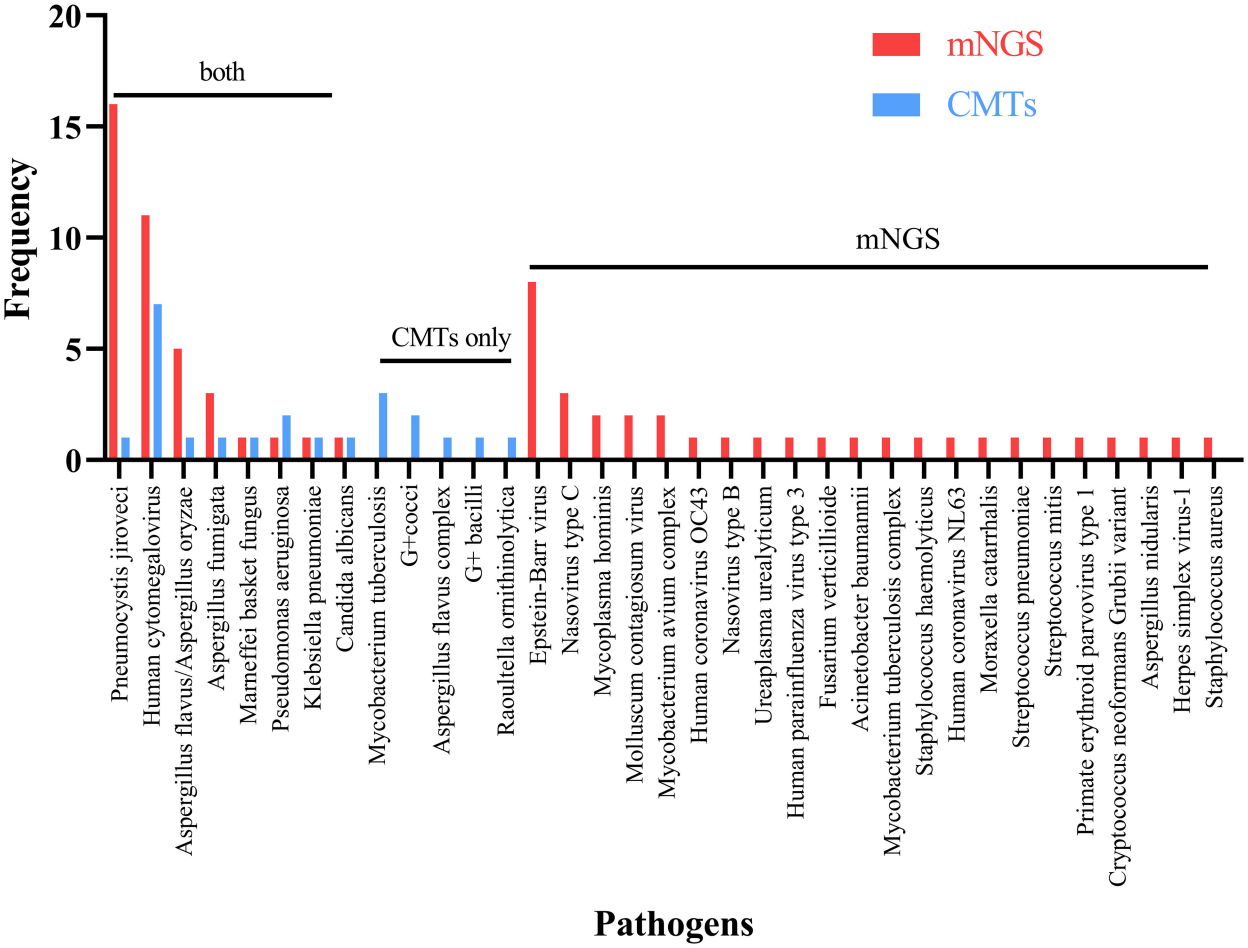
Breakdown of organisms identified by both metagenomic next generation sequencing (mNGS) and conventional microbiological tests (CMTs), mNGS only, and CMTs only.

In **Figure 2**, Pneumocystis jirovecii (16/74, 21.62%) was the most commonly detected pathogen through mNGS testing, followed by Human Cytomegalovirus (11/74, 14.86%), and Epstein-Barr virus (8/74, 10.81%). In contrast, Human Cytomegalovirus was identified as the most common pathogen detected through CMTs, with Mycobacterium tuberculosis as the second most prevalent pathogen. Eight common pathogens, namely Pneumocystis jirovecii, Human Cytomegalovirus, Aspergillus spp., Penicillium spp., Malassezia furfur, Pseudomonas aeruginosa, Klebsiella pneumoniae, and Candida albicans, were detected by both methods.

In this study, among the 36 infected individuals, 16 cases were tested positive for both mNGS and CMTs, 6 cases were negative in both methods, and 14 cases showed exclusive mNGS positivity. A statistically significant disparity in positivity rates between the two techniques within BALF samples was observed, with mNGS exhibiting a significantly higher rate compared to CMTs (83.3% VS 44.4%, P < 0.001).

#### 3.2.3 Concordance between mNGS and CMTs

Further comparison of the concordance between the two detection methods in identifying infectious pathogens revealed that 21 infected individuals displayed consistent results between the two methods (double-positive or double-negative). Among these, 6 infected individuals exhibited negative results in both methods, while among the 15 double-positive infected individuals, one showed complete concordance (complete overlap of all pathogens), 10 displayed partial concordance (one but not all pathogens overlapped), and 4 patients showed no concordance at all (no overlap of pathogens).

### 3.3 Relationship between CD4+ T lymphocyte counts and pathogen distribution detected by mNGS

Among PLHIV with CD4+T lymphocyte counts less than 50 cells/μL, 14 individuals were found to have two or more pathogens (co-infections), 3 PLHIV patients exhibited single-pathogen infections, and only 1 patient showed pathogen negativity. Among the 9 patients with CD4+T lymphocyte counts ranging from 50 to 200 cells/μL, 3 patients exhibited co-infections in the test results, 4 patients displayed single-pathogen infections, and 2 patients showed pathogen negativity. Among patients with CD4+T lymphocyte counts exceeding 200 cells/μL, 3 cases yielded pathogen-negative results, 4 cases had single-pathogen findings, and 2 cases displayed co-infections. The difference was statistically significant (χ2 = 6.645, P < 0.05). We proceeded with pairwise comparisons. The findings revealed that among individuals with multiple infections, the count of CD-positive T cells was significantly higher in those with <50/μl compared to those with >200/μl. However, no distinctions were observed among individuals with single infections and pathogen-negative individuals.

## 4 Discussion

In this study, both mNGS and CMTs were simultaneously employed to analyze BALF samples from 36 PLHIV, with a comparative assessment of the diagnostic performance of the two methods in detecting pathogens (bacteria, fungi, and viruses) within BALF samples.Firstly, mNGS yields results quickly, with the shortest reporting period compared to CMTs. Secondly, the findings of our study demonstrated a noteworthy superiority of mNGS over CMTs in terms of pathogen detection rate (83.33% VS 41.67%, P < 0.05). Another advantage of mNGS is its capability to detect a greater number of pathogens. Among the 36 PLHIV, mNGS identified a total of 30 pathogens, whereas CMTs detected only 13 pathogens.

Pneumocystis jirovecii is the most common opportunistic infection in PLHIV. Due to the lack of reliable *in vitro* culture methods, nucleic acid testing is commonly employed to detect Pneumocystis jirovecii (Shi et al., 2020). In this study, Pneumocystis jirovecii was the most commonly detected pathogen through mNGS (16/36, 44.44%), with only one case being detected via CMTs. The utilization of mNGS is expected to aid in the swift and precise detection of Pneumocystis jirovecii. Numerous studies have also confirmed the superiority of mNGS in pathogen identification (Wang et al., 2019).

Human Cytomegalovirus is a common opportunistic infectious pathogen that can infect the majority of the adult population. Although relatively harmless in healthy adults, Human Cytomegalovirus infection may result in serious end-stage conditions, including leukopenia, hepatitis, nephritis, interstitial pneumonia, gastrointestinal disorders, and, in some cases, fatality, particularly in individuals with compromised immune function or immunosuppression (Limaye and Boeckh, 2010; Yu et al., 2017; Wang et al., 2019). In our study, Human Cytomegalovirus emerged as the second most common pathogen detected through mNGS, with mixed infections of Human Cytomegalovirus and Pneumocystis jirovecii observed in 7 PLHIV. This finding is consistent with previous literature reports, which have observed CMV co-infections in patients infected with other pathogens, particularly Pneumocystis jirovecii (Miles et al., 1990; Salomon and Perlman, 1999; Benfield et al., 2001). Given the potential for severe consequences associated with Human Cytomegalovirus infection, rapid detection of CMV through mNGS is of paramount importance and facilitates early targeted antiviral therapy.

In addition, mNGS detected two or more pathogens in sputum cultures of 52.78% of patients, indicating the presence of multiple infections, which may be associated with the compromised immune function of PLHIV. Since CD4+T cells are closely associated with the body’s immune function (Fenwick et al., 2019), we further analyzed the association between CD4+T cells and multiple infections. We observed that as CD4+T cell counts declined, the likelihood of multiple infections increased, especially when patients had CD4+T cells <50/μL. Among the 36 PLHIV, 16 had CD4+T cell counts <50/μL, and out of those, 14 patients were diagnosed with multiple infections. Especially noteworthy is the markedly elevated probability of pulmonary polymicrobial infections among individuals with CD4+T cell counts <50/μl compared to those with CD4+T cell counts >200/μl. These findings suggest that clinical practitioners should consider the possibility of multiple infections in patients with low CD4+T cell counts and, when necessary, reinforce combination therapy.

Our study has some strengths. We examined the use of mNGS in PLHIV who may have lung infections and found that mNGS outperformed CMTs. This could help in the development of subsequent guidelines for detecting lung infections in PLHIV. We identified 30 pathogens in 36 PLHIV and provided a detailed proflie of these infections. Additionally, we investigated the distribution of pathogens in patients with varying CD4+T lymphocyte counts and discovered that PLHIV with a CD4+T lymphocyte count of less than 50 cells/μL had significantly higher infection rates compared to those with a higher CD4+T lymphocyte count. Some limitations should be acknowledged. The limited sample size in this study may have the potential to impact the accuracy of mNGS performance evaluation. And, due to the inherent uncertainties associated with laboratory procedures, we cannot definitively ascertain the absence of contamination in our samples. Nevertheless, we implemented several measures to mitigate contamination risks during our experimental procedures. These measures included aseptic techniques, regular cleaning and disinfection protocols, separate handling of samples, the use of reagents and consumables meeting quality control standards, and pre-training of laboratory personnel. As a result of these precautions, we have confidence in the relative validity of our findings. In addition, although our research indicates that mNGS significantly outperforms CMTs in detecting pathogens among PLHIV with suspected pulmonary infections, enabling rapid and comprehensive pathogen identification, facilitating timely and appropriate treatment strategies, and thereby potentially reducing unnecessary antibiotic usage and shortening hospital stays, there are also some challenges that cannot be overlooked. For instance, difficulties in distinguishing between infection and colonization pose a challenge, as we struggle to differentiate the pathogenicity status of pathogens through mNGS. Additionally, the relatively high cost of testing limits the widespread clinical application of mNGS.

Our study demonstrated a noteworthy superiority of mNGS over CMTs in terms of pathogen detection rate for PLHIV with suspected pulmonary infections. mNGS is capable of rapidly and accurately identifying the pathogen causing the pulmonary infection, which contributes to implement timely and accurate anti-infective treatment.

## Data availability statement

The datasets presented in this study can be found in online repositories. The names of the repository/repositories and accession number(s) can be found below: https://www.ebi.ac.uk/, PRJEB73333.

## Ethics statement

The studies involving humans were approved by The Ethics Committee of the sixth people hospital of Zhengzhou. The studies were conducted in accordance with the local legislation and institutional requirements. The participants provided their written informed consent to participate in this study. Written informed consent was obtained from the individual(s) for the publication of any potentially identifiable images or data included in this article.

## Author contributions

MH: Formal analysis, Methodology, Writing – original draft, Writing – review & editing. YW: Formal analysis, Methodology, Writing – original draft, Writing – review & editing. HY: Writing – review & editing. YZ: Writing – review & editing. XL: Writing – review & editing. NX: Writing – review & editing. QZ: Supervision, Writing – review & editing.
